# Who applies and who gets admitted to UK graduate entry medicine? - an analysis of UK admission statistics

**DOI:** 10.1186/1472-6920-11-71

**Published:** 2011-09-26

**Authors:** Paul Garrud

**Affiliations:** 1 School of Graduate Entry Medicine & Health, University of Nottingham, Royal Derby Hospital, Uttoxeter Road, DERBY DE22 3DT, UK

## Abstract

**Background:**

Graduate-entry medicine is a recent development in the UK, intended to expand and broaden access to medical training. After eight years, it is time to evaluate its success in recruitment.

**Objectives:**

This study aimed to compare the applications and admissions profiles of graduate-entry programmes in the UK to traditional 5 and 6-year courses.

**Methods:**

Aggregate data on applications and admissions were obtained from the Universities and Colleges Admission Service covering 2003 to 2009. Data were extracted, grouped as appropriate and analysed with the Statistical Package for the Social Sciences.

**Results:**

Graduate-entry attracts 10,000 applications a year. Women form the majority of applicants and admissions to graduate-entry and traditional medicine programmes. Graduate-entry age profile is older, typically 20's or 30's compared to 18 or 19 years in traditional programmes. Graduate-entry applications and admissions were higher from white and black UK ethnic communities than traditional programmes, and lower from southern and Chinese Asian groups. Graduate-entry has few applications or admissions from Scotland or Northern Ireland. Secondary educational achievement is poorer amongst graduate-entry applicants and admissions than traditional programmes.

**Conclusions:**

Graduate-entry has succeeded in recruiting substantial additional numbers of older applicants to medicine, in which white and black groups are better represented and Asian groups more poorly represented than in traditional undergraduate programmes.

## Background

Graduate entry medicine began in the UK in 2000 with the establishment of two programmes at St. Georges, London and a new medical school Leicester-Warwick. Since then, fourteen other courses have started - two in 2008. Graduate-entry programmes now account for 10% of admissions to medical school with 894 places in 2009-10. This development in the UK was an attempt to expand medical training numbers beyond the traditional A level school leaver population, to broaden access to the profession and to train doctors in four rather than five, or six, years [1,2].

At this point it is pertinent to review the profile of applicants and the success of recruitment to graduate entry medicine, in comparison to the established five and six year undergraduate courses.

## Methods

Data on applications and acceptances were obtained from the UK Universities and Colleges Admissions Service (UCAS) for A100 (twenty-six 5-yr or 6-yr medicine programmes) and A101 (graduate-entry) courses (fifteen 4-yr graduate-entry programmes - omitting Kings College, London - 24 places per annum - whose data is coded A104 by UCAS and confounded with other non-graduate-entry medicine programmes), covering the years 2003 to 2009, the period when all the current graduate-entry programmes have been in operation [3]. Data comprised aggregated frequencies for UCAS code A100 and A101 medicine courses, broken down by age band, gender, ethnicity, region of domicile and tariff band. Ethical approval was not sought since the UCAS data are anonymised and aggregated, hence fully protecting individual applicant confidentiality.

Ethnicity is self-declared on the UCAS application, but only for UK domiciled applicants. Tariff score is an aggregate points score of all UK secondary education level qualifications: it does not include any information about tertiary (higher) education achievement.

Data were extracted, grouped into a smaller number of composite categories for ethnicity, and analysed using simple summary measures. Statistical confirmation used the Chi square test.

## Results

### Applications

The number of graduate-entry applications increased from 2003 to 2004 and then stabilised around 10,000 per annum, about one fifth of the number of 5-year A100 applications. Women formed a significant majority in both sets of applications - graduate-entry (56.3%); A100 (56.4%), but the difference between their representation was not significant (p = 0.79).

Age profiles differ substantially since graduate-entry applicants have already completed a degree: 42.8% were aged 22-24 and 37.8% aged 25-39, only 2.8% aged 40 or more. Ethnicity profile differed significantly in terms of the proportions of applications: 63.9% vs. 60.4% white applicants to graduate-entry and A100 courses, 14.3% vs. 18.3% southern Asian, 5.6% vs. 8.0% Chinese Asian, and 8.2% vs. 5.1% black.

Reliable difference in domicile was evident: there were markedly fewer graduate-entry applicants than A100 from Scotland (2.6% vs 7.4%) and Northern Ireland (1.3% vs 4.3%), perhaps because no graduate-entry courses are available there, and fewer overseas applicants (2.7% vs 7.8%) - most UK graduate-entry courses offering places only to Home students. There were substantially more applicants from London (26% vs 18.5%). Secondary educational achievement (UCAS Tariff) was significantly weaker amongst applicants to graduate-entry courses, though this data was unavailable for many more (58%) graduate-entry applicants than A100 (12.2%).

A summary of the data and statistical analyses is shown in Table 1.

**Table 1:** Characteristics of applications and admissions to UK medical school 2003-9

	*Applications*			*Acceptances*		
	***A100***	***A101***	***Statistical significance***	***A100***	***A101***	***Statistical significance***

**Gender**						
male	149,296 (43.6%)	29,962 (43.7%)	n.s.	17,400 (42.1%)	2,343 (43.0%)	n.s.
female	193,005 (56.4%)	38,647 (56.3%)		23,911 (57.9%)	3,110 (57.0%)	

**Age**						
17-21	294,458 (86.0%)	11,423 (16.8%)		36,890 (89.3%)	969 (17.7%)	
22-24	25,888 (8.0%)	29,383 (42.8%)		2,539 (6.1%)	2,330 (42.7%)	
25-39	20,561 (6.0%)	25,903 (37.8%)	<0.001	1,797 (4.3%)	2,067 (37.9%)	<0.001
> = 40	1,394 (0.4%)	1,900 (2.8%)		85 (0.2%)	87 (1.6%)	

**Ethnicity**						
UK White	163,994 (60.4%)	38,820 (63.9%)		25,101 (68.2%)	3,949 (76.5%)	
Black - Caribbean, African & other	13,826 (5.1%)	5,004 (8.2%)		860 (2.3%)	260 (5.0%)	
Asian - Indian, Pakistani, Bangladeshi	49,660 (18.3%)	8,668 (14.3%)		5,609 (15.2%)	435 (8.4%)	
Asian - Chinese & other	21,730 (8.0%)	3,423 (5.6%)	<0.001	2,598 (7.1%)	183 (3.5%)	<0.001
Mixed	10,091 (3.7%)	2,169 (3.6%)		1,292 (3.5%)	188 (3.6%)	
Other & unknown	12,099 (4.5%)	2,703 (4.4%)		1,328 (3.6%)	146 (2.8%)	

**Domicile**						
North	46,670 (14.4%)	9,989 (14.9%)		6,126 (15.5%)	809 (15.1%)	
Midlands	56,775 (17.6%)	14,101 (21.1%)		7,609 (19.2%)	1,224 (22.8%)	
South	57,568 (17.8%)	14,016 (21.0%)		8,025 (20.3%)	1,313 (24.4%)	
London	60,032 (18.6%)	17,374 (26.0%)	<0.001	6,542 (16.5%)	1,397 (26.0%)	<0.001
Wales	12,278 (3.8%)	2,611 (3.9%)		1,770 (4.5%)	264 (4.9%)	
Scotland	24,085 (7.4%)	1,747 (2.6%)		4,154 (10.5%)	108 (2.0%)	
N.Ireland	13,857 (4.3%)	860 (1.3%)		2,553 (6.4%)	44 (0.8%)	
EU	26,992 (8.3%)	4,377 (6.5%)		993 (2.5%)	169 (3.1%)	
Overseas	25,247 (7.8%)	1,781 (2.7%)		1,849 (4.7%)	43 (0.8%)	

**Tariff**						
0-359	44,339 (18.5%)	16,851 (67.7%)		2,312 (6.8%)	1,275 (54.7%)	
360-419	45,103 (18.9%)	3,886 (15.0%)		4,909 (13.3%)	439 (18.8%)	
420-479	53,790 (22.6%)	2,341 (9.0%)	<0.001	8,235 (24.4%)	291 (12.5%)	<0.001
> = 480	95,083 (39.9%)	2,143 (8.2%)		18,286 (54.2%)	327 (14.1%)	

### Accepted applicants

Over this 7-year period 5,453 students were accepted onto graduate-entry courses. The proportions of women accepted were marginally higher than the proportions applying at 57.0% and 57.9% in both graduate-entry and A100, but were not significantly different. The age profile of graduate-entry acceptances was close to the applicant profile - 42.7% aged 22-24, 37.9% 25-39, 1.6% aged 40 or over. White students formed a larger proportion of acceptances (76.5%) in graduate-entry programmes than for A100 (68.2%), with smaller proportions of southern Asians (8.4% vs. 15.2%) and Chinese Asians (3.5% vs. 7.1%), but a higher proportion of black students (5.0% vs. 2.3%): this ethnicity profile, for graduate entry, was markedly and significantly different from A100. As with applications, few graduate-entry acceptances were from Scotland (2.0% vs. 10.5%), Northern Ireland (0.8% vs. 6.4%) or overseas (0.8% vs. 4.7%), but many more (26.0% vs. 16.5%) from London. Tariff scores, where available, amongst graduate-entry acceptances were lower than A100 - 73.5% graduate-entrants scoring below 420 cf. 78.6% A100 scoring 420 or higher - but higher than tariff scores amongst graduate-entry applicants (p < 0.001). Summaries of these data are also shown in Table 1.

### Selection ratio

The overall graduate-entry selection ratio (applications to admissions; UK applicants may apply to 4 separate medical schools) was 12.6:1, significantly higher than A100 (8.2:1) (p < 0.001), although this clearly varies substantially between medical schools, with higher ratios in London [4]. Selection ratios for subgroups therefore cannot be directly compared between graduate-entry and A100 courses. However, for graduate-entry courses it is clear that women applicants are more likely to be accepted than men, and white applicants than black or Asian. In comparison with A100 courses, graduate-entry selection ratios are comparable between black and Asian ethnicity applicants. The selection ratio information is summarised in Table 2.

**Table 2:** Acceptance to UK medical school 2003-9 as a function of gender and ethnicity

	*5/6 year programmes (UCAS code A100)*		*Graduate entry 4 year programmes (UCAS code A101)*	
	***Selection ratio***	***Standardised admission ratio***	***Selection ratio***	***Standardised admission ratio***

**Gender**				
male	8.5	0.80	12.7	0.84
female	8.0	1.21	12.4	1.15

**Ethnicity**				
UK White	6.5	0.77	9.8	0.85
Black - Caribbean, African & other	16.1	0.99	19.3	1.95
Asian - Indian, Pakistani, Bangladeshi	8.9	2.76	19.9	1.87
Asian - Chinese & Other	8.4	5.70	18.7	3.04
Mixed	7.8	1.79	11.5	3.17
Other & unknown	9.1	-	18.5	-

Selection ratio: no. applications per acceptance

Standardised admission ratio: proportion of acceptances ÷ proportion in UK population (2001 census)

## Discussion

Graduate entry programmes have been successful in attracting a considerable boost in applications to medicine in the UK. This has not reduced the numbers applying to traditional 5/6-year courses; indeed they have also increased, from 8,108 Home applicants in 2000 to 13,468 in 2009.

Women are more likely to be applicants and to be accepted for graduate entry than men, as is the case for traditional school leaver courses. The typical graduate-entry student started their course in their mid-20's although a small proportion were in their 30's and a very few in their 40's: the length and quality of their clinical service will be important outcomes to evaluate in the future.

So far, graduate-entry medicine appears to be attracting and selecting higher proportions of students from white and black ethnic groups than from Asian communities in the UK, compared to the traditional 5/6 year courses. Whilst this suggests examination of the selection criteria and tools used, it is also worth considering the balance of applications for medicine in terms of the representation of different ethnic communities in the UK population. One recent study [5] noted that standardised admission ratios (number admitted to medical school as a proportion of the number if places were allocated equitably across all ethnic groups) were 6.07 (over-represented) amongst UK Asians compared to 0.73 (under-represented) in white students in 2000; Table 2 also provides that data for 2003-2009 admissions to A100 and graduate-entry courses.

The evidence about prior educational achievement is limited, but suggestive. For graduate-entry courses secondary educational attainment is poorer on average than for traditional 5/6-year programmes, but each graduate-entry applicant and student has also demonstrated achievement in tertiary education.

One recent study [6] that investigated medical school intake in UK 2002-6, found very similar patterns in terms of age, gender and ethnicity to those reported in the present study for the period 2003-9. In addition that study reported that graduate entry programmes were significantly less likely to admit students from higher professional and managerial parental backgrounds (Odds Ratio 0.6).

The UK pattern reported here - of rising applications, expanding entrant numbers, and a majority of women - contrasts with the situation internationally. In USA, women comprise just under 50% of applicants and enrolments and this proportion has been stable from 2001-8; applicant and enrolment numbers have scarcely changed over 25 years, being stable around 17,000 per annum [7]. In Australia, the proportion of men to women has also approximated 50:50 since the 1990s and this has remained stable despite the introduction of graduate-entry and the doubling of medical schools and places since 2000 [8]. In other European countries, there is divergence between the Western and former Eastern bloc in terms of gender: a clear majority of women entering medicine in former Eastern bloc countries, but a minority, albeit approaching 50%, in most Western countries [9].

The other germane international perspective is the supply of doctors compared to the national population. In 2005, there were 2.1 physicians per 1,000 people in UK, but an average 3.4 across all the other European countries [10]. The UK has been a marked importer of doctors for many years (as also is USA), compared to other European countries [11], but the doubling of places in UK medical schools since 1997 and the introduction of graduate-entry is likely to reduce the difference in physician supply and may also decrease the proportion of international medical graduates practising in the UK.

This picture of who applies and who gets admitted to UK graduate-entry courses is a composite one, but a more detailed analysis awaits collaborative research between the diverse graduate-entry schools.

## Conclusions

Graduate-entry in the UK has succeeded in recruiting substantial additional numbers of older applicants to medicine, in which white and black groups are better represented and Asian groups more poorly represented than in traditional undergraduate programmes.

## Competing interests

The author declares that they have no competing interests.

## Authors' contributions

The author is solely responsible for all aspects of this study.

## Author's information

The author works as Assistant Director of Medical Education at the University of Nottingham School of Graduate Entry Medicine & Health, where he is the Academic Lead for admissions.

## Pre-publication history

The pre-publication history for this paper can be accessed here:

http://www.biomedcentral.com/1472-6920/11/71/prepub
